# *In vivo* three-dimensional structural analysis of cilia by cryo-electron tomography

**DOI:** 10.1186/2046-2530-1-S1-P25

**Published:** 2012-11-16

**Authors:** T Ishikawa, KH Bui, T Movassagh, G Pigino, A Maheshwari

**Affiliations:** 1 Paul Scherrer Institute, Switzerland

## 

Ciliary bending motion is driven by sliding of microtubule doublets with dynein molecular motors. Dyneins form two complexes: inner and outer dynein arms. The inner arm is supposed to determine the waveform, while the outer arm is likely an accelerator. However, the mechanism to integrate linear motion of dynein into well-orchestrated axonemal bending is still to be investigated. We are analyzing 3D structure of cilia from *Tetrahymena*, *Chlamydomonas*, sea urchin sperm and mouse trachea, to reveal the novel architecture of cilia. By comparing 3D structure of *Chlamydomonas* mutant axonemes, we located all the three outer arm dyneins and the eight inner arm dyneins (Bui et al. (2008) J. Cell Biol. 183, 923). Dyneins, which consist of the N-terminal tail, the ATPase ring, the stalk and the microtubule binding domain, undergo conformational change during the phosphate release: the ATPase ring shifts 8nm toward the distal end, which can explain the minus-end driven motor activity of dynein. Interestingly, *in vivo*, some dyneins change their conformations, while the other stay in the apo form, suggesting negative cooperativity among dyneins and bending mechanism caused by torsion at the boundary between dynein in the apo form and in the nucleotide-binding form (Movassagh et al. (2010) Nat. Struct. Mol. Biol. 17, 761). We also reconstructed 3D structure of the radial spoke, regulatory complex of 23 proteins (Pigino et al. (2011) J. Cell Biol. 195, 673). Each radial spoke has pseudo-two fold symmetry, indicating dimeric nature. Our 3D structure implies the assembly pathway of the radial spoke.

http://www.psi.ch/lbr/takashi-ishikawa

